# Pre-operative neutrophil count and neutrophil-lymphocyte count ratio (NLCR) in predicting the histological grade of paediatric brain tumours: a preliminary study

**DOI:** 10.1007/s00701-017-3388-5

**Published:** 2017-11-29

**Authors:** J. R. F. Wilson, F. Saeed, A. K. Tyagi, J. R. Goodden, G. Sivakumar, D. Crimmins, M. Elliott, S. Picton, P. D. Chumas

**Affiliations:** 1 Department of Neurosurgery, The General Infirmary at Leeds, Leeds, LS13EX UK; 2 Department of Neurosurgery, Beaumont Hospital, Dublin, Ireland; 3 Department of Paediatric Oncology and Haematology, Leeds Children’s Hospital, Leeds General Infirmary, Leeds, LS13EX UK

**Keywords:** Paediatric, Brain tumour, Neutrophil lymphocyte, Grade

## Abstract

**Introduction:**

The neutrophil-lymphocyte count ratio (NLCR) is an established prognostic marker for renal, lung and colorectal carcinomas and has been suggested to be predictive of histological grade and outcome in adult intracranial tumours. The purpose of this study was to determine whether a correlation of the pre-operative neutrophil count (NC) and NLCR with the final histological grade exists in paediatric intracranial tumours.

**Methods:**

A retrospective analysis was undertaken at a single centre. Patients less than 18 years old at the time of surgery who underwent tumour-related procedures from 2006 to 2015 were included. Patients with recurrent tumours, previous bone marrow transplant and metastases were excluded. Pre-operative full blood counts (FBC), collected before the diagnosis of intracranial pathology and before administration of steroids, were matched with histological diagnosis for each patient. Post-operative FBC was also recorded, together with survival data where applicable.

**Results:**

A total of 116 patients (74 male, 42 female; mean age, 8 ± 0.9 years) with a diagnosis of primary intracranial tumours had pre-operative FBC that could be matched to final histological grade. Pre-operative NC and NLCR were higher with increasing grade of tumour: grade 1 (NC 4.29 10^9^/l, NLCR 2.26), grade 2 (NC 4.59 10^9^/l, NLCR 2.38), grade 3 (NC 5.67 10^9^/l, NLCR 2.72) and grade 4 (NC 6.59 10^9^/l, NLCR 3.31). Patients with WHO grade 1 and 2 tumours pooled together had a lower NC (4.37 95% CI ± 0.67 10^9^/l) compared to WHO grade 3 and 4 patients (6.41 95% CI ± 0.99 10^9^/l, *p* = 0.0013). The NLCR was lower in grade 1 and 2 tumours (2.29 ± 0.59) (compared to grade 3 and 4 tumours; 3.20 ± 0.76) but this did not reach significance (*p* = 0.069). The subgroup of patients with pilocytic astrocytoma had a significantly lower NC when compared to patients with high-grade tumours (*p* = 0.005). Medulloblastoma and supratentorial PNET subgroups had significantly higher NC compared to the low-grade group (*p* = 0.033, *p* = 0.002). Post-operative NC was significantly higher in the high-grade tumours (*p* = 0.034), but no difference was observed for NLCR (*p* = 0.28).

**Conclusions:**

No evidence exists to support the correlation of pre-operative NC or NLCR to histological diagnosis in paediatric intracranial tumours. Our results indicate that a higher pre-operative NC/NLCR correlates with a higher histological grade of tumour. This suggests that immunological mechanisms may be involved in the pathogenesis of paediatric brain tumours, and a further prospective study is required to substantiate and expand these findings.

## Introduction

One hundred fifty years ago Virchow suggested that cancer derived from sites of chronic inflammation [1]. In recent years Virchow’s hypothesis has borne true, and the association between inflammation and the pathogenesis of certain neoplasms (e.g papillomavirus in cervical cancer) has been demonstrated. Examination of the microenvironment of tumours reveals that host leucocytes, including tumour-associated macrophages and lymphocytes, are deeply involved in the pathophysiology of neoplasms [13, 16]. Indeed, potentiation of cellular activation and signalling mechanisms is responsible for angiogenesis, production of pro-inflammatory cytokines/chemokines, tumour growth and destruction of the extracellular matrix [1, 9, 13]. Tumour-necrosis factor (TNF), interleukins 1, 4, 5 and 10, and interferon gamma are some of the many notable messengers in this pathway that have been the focus of research into the design of anti-neoplastic and auto-immune therapies [4, 15]. More recent work has shown tumour-associated neutrophils play little role in host defence, and modulating/down-regulating their activation can significantly restrict tumour growth in animal models [4, 6]. It has also been postulated that the severity of the inflammatory response in neoplastic processes may correlate with tumour progression/grade of tumour [8, 11].

Given the inherently nonspecific nature of the inflammatory cascade, it follows that sites of inflammation/tumorogenesis will produce a response that can be detected systemically [12]. This response not only causes an increase in the NC, but also serves to promote margination and apoptosis of leucocytes, which could lead to an increase in the NLCR [3].

Measurements of the NC and NLCR in peripheral blood have been shown to be an important prognostic marker in a number of different malignancies [7], with thresholds predictive of a poor prognosis established for many of these. A recent retrospective analysis of 1300 oncology patients correlated an elevated NLCR of 3 or more with a significantly reduced overall survival [7]. Schmidt et al. in 2005 [15] demonstrated that monocyte elevation, in addition to neutrophil elevation, was also correlated with a poorer prognosis in patients with metastatic melanoma.

Peripheral blood counts in patients with brain tumours are not normal when compared to healthy controls [17]. This is unusual, as the brain is traditionally considered an immune-privileged site and central nervous system inflammation/infection can often manifest with little systemically measurable response [10]. Several studies have been published suggesting that the relationship between NLCR and the histological grade of tumours can be demonstrated in adult brain tumours [2, 5, 19]. Han et al. [5] showed that for adult glioblastoma patients an NLCR greater than 4 was associated with worse 2-year survival. Zadora et al. [19] stratified adult patients with grade 1–4 glial tumours to demonstrate that an NLCR of greater than 2.579 was significantly associated with a high likelihood of predicting glioblastoma. Bambury et al. conducted multivariate analysis on glioblastoma patients to investigate the correlation with survival [2]. Patients with an NCLR of < 4 had a median survival of 4 months longer (11.2 months) compared to those with an NLCR > 4 (7.5 months), independent of other known prognostic factors. An NLCR > 4 has also been demonstrated as a prognostic indicator of poor survival in recurrent glioblastoma [14].

Recently, Tumturk et al. [18] have shown that a higher mean NLCR, mean platelet volume and mean white cell count may correlate with the presence of an intracranial tumour in children less than 3 years old (compared to age-matched controls). However, to date no study has been published looking at paediatric brain tumours to distinguish whether pre-operative neutrophils or the NLCR are associated with histological grading. Our hypothesis was that the association between high pre-operative neutrophils/NLCR and higher histological grading demonstrated in adults was also present in paediatric brain tumours.

## Materials and methods

A retrospective analysis of the paediatric operative database was performed at a single centre in the Department of Neurosurgery, Leeds. The database was interrogated to identify all patients under the age of 18 years old (at the time of surgery) who underwent tumour-related procedures over 10 years (2006–2015). Patients were excluded from the final analysis if surgery was for recurrent tumours or metastases, if patients had had a previous bone marrow transplant or if verified pre-operative FBC data were not available. Patients with a histological diagnosis of craniopharyngioma were excluded as this was considered an extra-axial tumour for the purposes of this study.

The time- and date-matched pre-operative FBC for each patient was recorded. Patient drug administration records were interrogated to ensure the FBC was taken prior to the first administration of steroids or the FBC was confirmed to have been taken prior to the first patient imaging (and thus prior to diagnosis) to eliminate any effect of steroids on the subsequent NLCR. Each patient was then matched to the subsequent histological diagnosis (and grading), and the NLCR was calculated for each. The post-operative NLCR was also collected.

The data were analysed using SPSS Statistics, version 22.0 (IBM, Chicago, IL). The mean and confidence intervals were measured for normally distributed data and one-way analysis of variance (ANOVA) was used to determine the difference between and within the groups, with Student’s two-tailed t test used to compare means between subgroups. Grouped means of the NC or NLCR, or by histological diagnosis, were calculated with 95% confidence intervals. The results were considered to be statistically significant when the *p* value was less than 0.05. Receiver-operator characteristic (ROC) curves with auto-calculated maximum specificity and sensitivity were constructed and 95% confidence intervals used for calculation of the area under the curve. The Youden Index was used to calculate cut-off points for NC and NLCR values.

## Results

Two hundred fourteen patients underwent procedures for intracranial tumours over the 10-year period interrogated; 116 patients (42 female, 74 male) underwent primary procedures with verified pre- and post-operative FBCs and a subsequent histological diagnosis. Thirty-nine patients were diagnosed with grade 1 tumours, 14 with grade 2, 12 with grade 3 and 51 with grade 4. The distribution of all histological subtypes for all patients included is shown in Table 1. Medulloblastoma (*n* = 25) and pilocytic astrocytoma (*n* = 23) were the most common tumour types, followed by supratentorial PNET (*n* = 12).

**Table 1:** Number of patients by histological diagnosis

**Tumour histological diagnosis**	**n**
Pilocytic astrocytoma	23
Non-metastatic medulloblastoma	16
Supratentorial primitive neuroectodermal tumour (PNET)	12
Metastatic medulloblastoma	9
Glioblastoma	7
ATRT	5
Anaplastic ependymona	5
Diffuse astrocytoma	5
Dysembrioplastic neuroepithelial tumour (DNET)	5
Ganglioglioma	5
Anaplastic astrocytoma	4
Subependymal giant cell histiocytoma (SEGA)	3
Anaplastic oligodendroglioma	2
Central neurocytoma	2
Oligodendroglioma	2
Pilomyxoid astrocytoma	2
Low-grade non-specific	1
Atypical ganglioglioma	1
Choroid plexus papilloma	1
Desmoplastic infantile ganglioglioma	1
Ependymoblastoma	1
Ependymoma	1
Gangliocytoma	1
Papillary tumour of the pineal region	1
Pineoblastoma	1

**Fig. 1:**
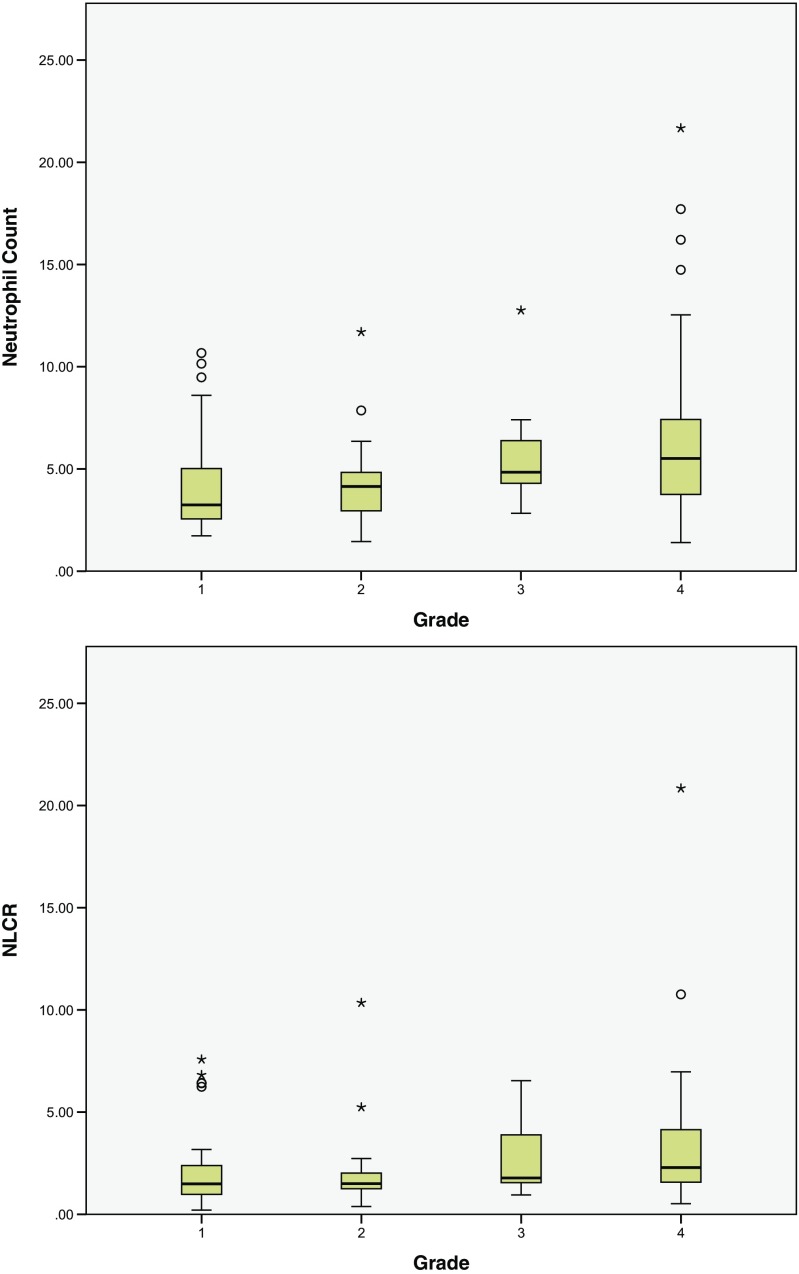
Box plots of the neutrophil count and neutrophil-lymphocyte count ratio by tumour grade. Thick black line, median value; tan box, 25th–75th quartile range; thin bars, range of values excluding outliers

The pre-operative NCs for grade 1 and 2 tumours were 4.29 (95% CI ± 0.77) 10^9/land 4.59 (95% CI ± 1.39) 10^9/l respectively, which was lower than for grade 3 and 4 tumours (5.67 95% CI ± 1.48 10^9/l and 6.59 95% CI ± 1.17 10^9/l). A similar relationship was observed for the NLCR, with 2.26 (95% CI ± 0.65) and 2.38 (95% CI ± 1.37) for grades 1 and 2 and 2.72 (95% CI ± 1.03) and 3.31 (95% CI ± 0.91) for grades 3 and 4 respectively (Table 2). One-way ANOVA comparing the NC between grades shows a significant difference between groups (*p* = 0.011, Fig. 1), but this difference is not significant regarding NLCR (*p* = 0.289).

**Table 2:** Neutrophil count (NC) and neutrophil-lymphocyte count ratio (NLCR) for grade 1–4 tumours, “low-grade” versus “high-grade” tumours and by the most common tumour sub-types

	n	Neutrophils	Neutrophil-lymphocyte count ratio
Grade 1	39	4.29 ± 0.77	2.26 ±0.65
Grade 2	14	4.59 ± 1.40	2.38 ±1.37
Grade 3	12	5.67 ± 1.48	2.72 ±1.03
Grade 4	51	6.59 ± 1.17	3.31 ± 0.91
“Low grade” (1 and 2)	53	4.37 ± 0.67	2.29 ±0.59
“High grade” (3 and 4)	59	6.41 ± 0.99	3.20 ± 0.76
Pilocytic astrocytoma	23	3.98 ± 0.73	2.06 ± 0.66
Non-metastatic medulloblastoma	16	6.00 ± 1.84	2.74 ± 0.95
Metastatic medulloblastoma	9	4.41 ± 1.47	2.54 ± 1.11
Medulloblastoma (all)	25	5.79 ± 1.27	2.67 ± 0.72
Supratentorial PNET	12	7.76 ± 3.39	4.69 ± 3.35

**Fig. 2:**
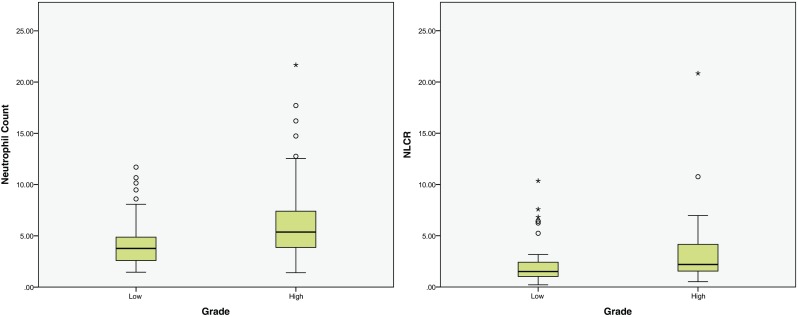
Box plots of the neutrophil count and neutrophil-lymphocyte count ratio of “low-grade” and “high-grade” tumours (see Fig. 1 legend for key)

The pooled samples of grade 1 and 2 tumour patients (“low grade”) had a significantly lower NC when compared to the grade 3 and 4 patients (“high grade”): 4.37 (95% CI ± 0.67) 10^9/l compared to 6.41 (95% CI ± 0.99) 10^9/l (*p* = 0.0013, Fig. 2). The NLCR of the low-grade group was also lower (2.29 ± 0.59) than that of the high-grade group (3.20 ± 0.76), which was not significant (*p* = 0.069). The average monocyte count across all patients was 0.45 ± 0.14, with no significant difference between the low- and high-grade groups (*p* = 0.45). Post-operative NC was significantly lower in the low-grade group (10.10) compared to the high-grade group (12.42, *p* = 0.034), whereas there were no significant differences in the post-operative NLCR (*p* = 0.28).

**Fig. 3:**
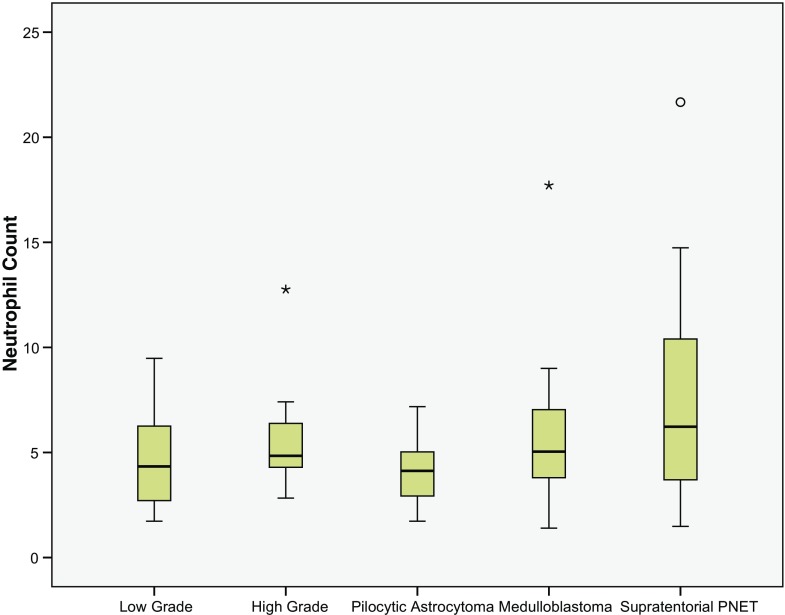
Box plots of the neutrophil count (NC) of the “low-grade” and “high-grade” subgroups together with the pilocytic astrocytoma, medulloblastoma and supratentorial PNET subgroups (see Fig. 1 legend for key)

**Fig. 4:**
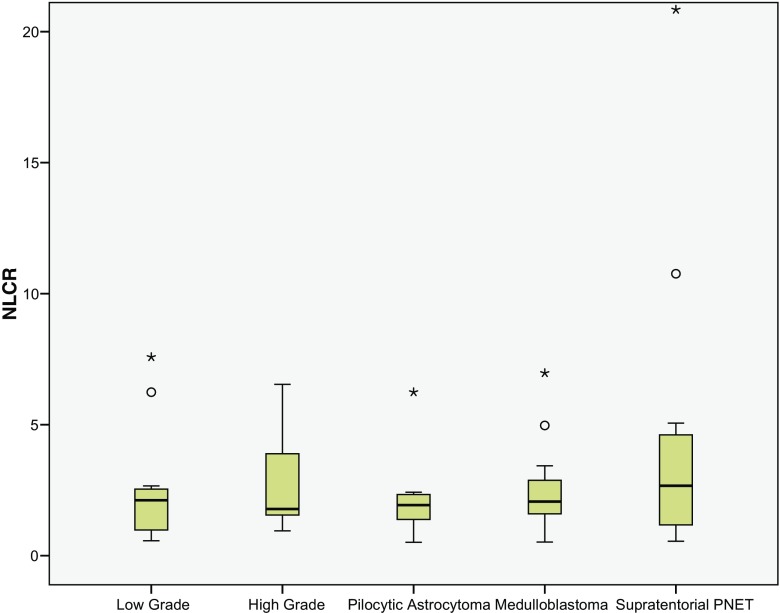
Box plots of the neutrophil-lymphocyte count ratio (NLCR) of the “low-grade” and “high-grade” subgroups together with the pilocytic astrocytoma, medulloblastoma and supratentorial PNET subgroups (see Fig. 1 legend for key)

Subgroup analysis was performed to compare the most common low-grade tumour type (pilocytic astroctyoma) to the “high-grade” group. The pre-operative NC was significantly lower (3.98 95% CI ± 0.73 10^9/l) compared to the high-grade tumours (*p* = 0.005, see Fig. 3). This difference was not significant regarding the NLCR (*p* = 0.09, see Fig. 4).

The most common high-grade tumours (medulloblastoma and supratentorial PNET) were also compared to the “low-grade” group. Both subgroups had a significantly higher pre-operative NC (5.79 95% CI ± 1.27 10^9/l, 7.76 95% CI ± 3.39 10^9/l) compared to the low-grade tumours (*p* = 0.033, *p* = 0.002, Fig. 3). The NLCR was also significantly higher in the supratentorial PNET group (4.69 95% CI ± 3.35) compared to the low-grade NLCR group (*p* = 0.019, Fig. 4), but this was not reflected in the medulloblastoma group (2.67 95% CI ± 0.72, *p* = 0.44). There was no difference in the pre-operative neutrophils or NLCR between metastatic medulloblastoma on presentation or non-metastatic on presentation (*p* = 0.67, *p* = 0.79).

**Fig. 5:**
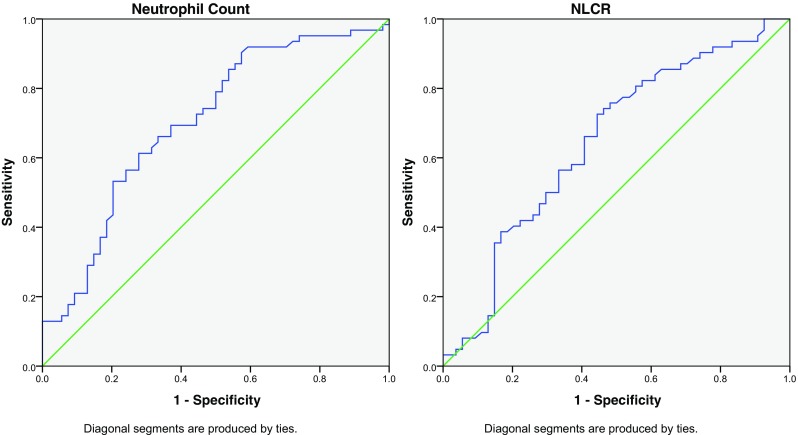
Receiver-operating characteristic (ROC) curve for neutrophil count and neutrophil-lymphocyte count ratio predicting the presence of a high-grade tumour

Receiver-operating characteristic (ROC) curve analysis was performed to evaluate the potential for the pre-operative NC or NLCR to be used to predict histological high-grade tumours (Fig. 5). The area under the curve for the NC was 0.709 (fair), with a cut-off value (using Youden’s Index) of 4.76 10^9/l. The area under the curve for NLCR was 0.637 (poor), with a cut-off value of 1.63.

**Fig. 6:**
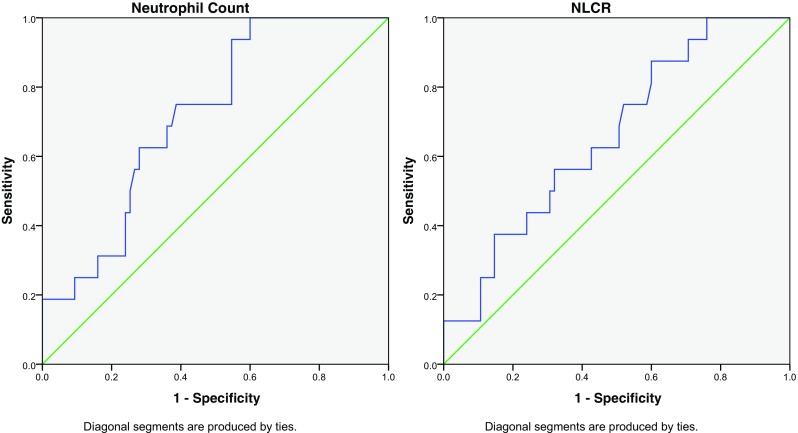
Receiver-operating characteristic (ROC) curve for neutrophil count and neutrophil-lymphocyte count ratio predicting death at 2-year follow-up

Thirty-one deaths were recorded: two patients from the grade 2 group, six from the grade 3 group and 23 from the grade 4 group. The mean overall survival for the patients who died in the grade 2 group was 52 months, with 18 and 15 months for the mortalities in the grade 3 and 4 groups respectively. ROC curve analysis was performed to determine whether the pre-operative NC or NLCR had a predictive value for death within 2 years (with medulloblastoma patients removed, Fig. 6). The analysis suggests a pre-operative NC higher than 3.36 and a NLCR higher than 1.96 would predict death at 2 years. This showed a reasonable correlation for the NC (0.724) according to the area under the curve, but a poor correlation for the NLCR (0.649). Four patients (one grade 3 and three grade 4) who died had either an NC or NLCR more than 2 standard deviations greater than the mean. Their pooled survival (8.8 months) was shorter compared to the mean survival of the other high-grade tumours (15.6 ± 4.8), but this was not statistically significant (*p* = 0.17). Of the 29 deaths in the high-grade group, 17 patients had a NLCR < 4 and 7 patients had a NLCR > 4. This gave an odds ratio (OR) of death within 2 years of high-grade patients with an NLCR > 4 of 0.95 (*p* = 0.93).

## Discussion

It is well established that the inflammatory cascade and immune response play a role in the initiation and propagation of metastatic and non-metastatic neoplasms [1, 9, 13, 16]. For a variety of tumours peripheral blood measurement of neutrophils, monocytes or the NLCR can provide correlation with prognosis or predict response to treatment [7, 12, 15]. Recent publications have suggested that a systemic response can even be measured when tumours are confined to immune-privileged sites such as the brain and can be correlated with the presence of malignant histological types and prognosis [5, 17, 19]. It would therefore follow that measurement of the systemic immune response should provide similar insights regarding paediatric intracranial tumours, albeit with a different spectrum of histological subtypes.

This study is the first we are aware of that analyses the significance of the pre-operative NC and NLCR with paediatric intracranial tumours. Our results show a significant increase in the mean pre-operative NC and NLCR with an increase in the WHO histological grade, which echoes the findings of previous studies in adult patients [2, 5, 19]. These findings expand those described by Tumturk et al. [18], which demonstrate children <3 years old with intracranial tumours have a higher WCC and NLCR when compared to age-matched controls. Pooled analysis of high-grade tumour patients demonstrates a significant increase in the pre-operative NC when compared to the low-grade patients (*p* = 0.0013), but this just failed to achieve significance with the NLCR (*p* = 0.069). The results reflect that this study is marginally under-powered to demonstrate (potentially) a true difference in the mean NLCR for these cohorts. These findings could be confounded by the increased chance that patients with high-grade lesions will have been administered steroids, which would account for a higher NC/NLCR. The authors have taken the necessary steps to remove this confounding effect as much as possible by the methods employed in the data collection; however, this potential confounder could be eliminated completely by a well-designed prospective study. The numbers of patients with grade 2 and 3 tumours are relatively low compared to those with grades 1 and 4. This is a reflection of the incidence of these tumour subtypes, and the study may be under-powered to truly represent these grades.

The pre-operative NC appears to produce a more discriminating receiver-operating characteristic (ROC) curve when compared to the NLCR in our data set. The Youden Index was used to provide cut-off threshold values, and interpretation of the area under the curve results was based on routine statistical standards. An NC of 4.76 10^9/l or higher appears to predict the presence of a high-grade histological diagnosis; however, given the mean NC for grade 1 tumour patients was 4.01, there is little basis for this to be recommended for potential prognostic use based on this evidence. Moreover, a more meaningful interpretation would be that the area under the curve of 0.709 certainly suggests that there is good evidence of a potentially useful correlation that may become apparent with a higher number of patients. Similarly, the NLCR cut-off value produced by ROC analysis (1.59) falls below the average NLCR for grade 1 patients and demonstrates a poor correlation (area = 0.637). This cut-off is lower than has been suggested in adults [19]; however, Zadora et al.’s suggested value of 2.579 was to predict the presence of glioblastoma and not just all high-grade tumours. This suggests that the true value of the NLCR to predict high-grade paediatric intracranial tumours may be lower than in adults. If this were true then the use of an NLCR cutoff value of 4 (used in the published adult studies [2, 5]) would not be applicable in the paediatric population. This could be potentially explained by the difference in distribution of histological diagnoses compared to the adult population. This is further borne out when you consider the use of an NLCR cut-off of > 4 in our data set did not produce an OR of death higher than the < 4 group. The use of ROC analysis to assess 2-year survival appeared to demonstrate a reasonable correlation with the NC, with a value of 3.36 or greater predictive of mortality. Medulloblastoma was removed from the survival analysis as, although histologically a high-grade tumour, this usually carries a 5-year survival rate of 75–80%, which is distinctly different from the survival rates of the other high-grade tumours represented and may have adversely confounded the analysis of the grade 4 tumours. The ROC analysis of NLCR showed a poor area under the curve, but suggested a trend toward a higher NLCR correlating with a higher risk of mortality at 2 years. The authors suggest the results of this study cannot be used to determine whether the NC or NLCR could predict 2-year mortality, but certainly are grounds to suggest a potential correlation that may have important clinical and therapeutic implications.

Subgroup analysis on the pilocytic astrocytoma patients showed significantly lower pre-operative NCs and lower (but not significantly) NLCR compared to high-grade tumours. There was a reciprocal result with medulloblastoma and supratentorial PNET with the pre-operative NC significantly higher than the low-grade tumours and also with the NLCR in the supratentorial PNET group. These findings seem to suggest that malignant or more aggressive tumours are more likely to elevate the systemic immune response, which potentially can be explained by increased destruction of the blood-brain barrier.

## Conclusions

This retrospective case series represents the largest number of paediatric patients with intracranial tumours that correlates the pre-operative NC and NLCR with histological WHO grading. There appears to be good evidence to suggest paediatric patients with a higher pre-operative NC or NLCR are more likely to have a higher WHO grade on histological diagnosis. This would support the notion that, despite the nervous system being an ‘immune-privileged’ site, disruption of the blood-brain barrier and immunological modulation may be an important factor in the development and potentiation of paediatric intracranial tumours.

This study adds to the previous literature describing the correlation between systemic elevation of the immune response and paediatric intracranial tumours. These preliminary findings should provide further justification for prospective investigations into the potential immunological pathogenesis of intracranial tumours in the paediatric population.
